# Neutrophils mediate early cerebral cortical hypoperfusion in a murine model of subarachnoid haemorrhage

**DOI:** 10.1038/s41598-019-44906-9

**Published:** 2019-06-11

**Authors:** Axel Neulen, Tobias Pantel, Michael Kosterhon, Andreas Kramer, Sascha Kunath, Maximilian Petermeyer, Bernd Moosmann, Johannes Lotz, Sven R. Kantelhardt, Florian Ringel, Serge C. Thal

**Affiliations:** 1 Department of Neurosurgery, University Medical Centre of the Johannes Gutenberg-University of Mainz, Langenbeckstrasse 1, 55131 Mainz, Germany; 2 Institute for Pathobiochemistry, University Medical Centre of the Johannes Gutenberg-University of Mainz, Duesbergweg 6, 55128 Mainz, Germany; 3 Institute for Clinical Chemistry and Laboratory Medicine, University Medical Centre of the Johannes Gutenberg-University of Mainz, Langenbeckstrasse 1, 55131 Mainz, Germany; 4 Department of Anaesthesiology, University Medical Centre of the Johannes Gutenberg-University of Mainz, Langenbeckstrasse 1, 55131 Mainz, Germany

**Keywords:** Stroke, Brain injuries

## Abstract

Cerebral hypoperfusion in the first hours after subarachnoid haemorrhage (SAH) is a major determinant of poor neurological outcome. However, the underlying pathophysiology is only partly understood. Here we induced neutropenia in C57BL/6N mice by anti-Ly6G antibody injection, induced SAH by endovascular filament perforation, and analysed cerebral cortical perfusion with laser SPECKLE contrast imaging to investigate the role of neutrophils in mediating cerebral hypoperfusion during the first 24 h post-SAH. SAH induction significantly increased the intracranial pressure (ICP), and significantly reduced the cerebral perfusion pressure (CPP). At 3 h after SAH, ICP had returned to baseline and CPP was similar between SAH and sham mice. However, in SAH mice with normal neutrophil counts cortical hypoperfusion persisted. Conversely, despite similar CPP, cortical perfusion was significantly higher at 3 h after SAH in mice with neutropenia. The levels of 8-iso-prostaglandin-F2α in the subarachnoid haematoma increased significantly at 3 h after SAH in animals with normal neutrophil counts indicating oxidative stress, which was not the case in neutropenic SAH animals. These results suggest that neutrophils are important mediators of cortical hypoperfusion and oxidative stress early after SAH. Targeting neutrophil function and neutrophil-induced oxidative stress could be a promising new approach to mitigate cerebral hypoperfusion early after SAH.

## Introduction

Aneurysmal subarachnoid haemorrhage (SAH) is one of the most common forms of haemorrhagic stroke^1–3^. Frequently the neurological outcome is poor, especially in cases presenting with coma and focal deficits. Morbidity and mortality may arise from the haemorrhage itself, which causes a dramatic increase in intracranial pressure (ICP). Even in patients surviving early ICP elevation, however, brain damage develops in two distinct phases termed early brain injury (EBI)^4,5^ and delayed cerebral ischemia (DCI)^4,6^. Early brain injury is caused by transient global cerebral ischemia during the haemorrhage and by toxic processes induced by the subarachnoid haematoma^4–6^. Conversely, DCI occurs days and weeks after SAH from the combined effects of vasospasm in large intracranial vessels and microvessels, microthrombosis and ischemia related to cortical spreading depression, and proinflammatory processes triggered by EBI^4,6,7^.

While major research efforts over the past few decades have focused on DCI (reviewed in^6^), recent clinical research data indicate that the degree of cerebral hypoperfusion during the first hours after SAH determines the severity of EBI, the occurrence of DCI, and the neurological outcome^4,8–14^. These findings suggest that EBI-associated processes causing cerebral hypoperfusion early after SAH are critical determinants of DCI severity and neurological outcome. A more complete understanding of processes leading to early cerebral hypoperfusion following SAH could therefore facilitate the development of new therapeutic strategies.

Blood in the subarachnoid space and subsequent haemoglobin degradation trigger an inflammatory cascade^7,15^. The central role of leukocyte-dependent inflammation in the etiology of delayed cerebral vasospasm was demonstrated by results showing attenuated delayed vasospasm following experimental SAH by blocking leukocyte extravasation with antibodies directed against intercellular adhesion molecule 1, cluster of differentiation 11 (CD11), or CD18^16,17^. Furthermore, induction of neutropenia by injection of an anti-Ly6G antibody attenuated delayed cerebral vasospasm^18^ and improved neurological outcome^19^. In line with these experimental data, a high percentage of neutrophils in human cerebrospinal fluid (CSF) samples on day 3 post-SAH was associated with development of cerebral vasospasm and markers of oxidative stress were elevated in CSF samples from SAH patients^20^.

However, neutrophils accumulate at the site of infection within minutes^21–23^. Similarly, Friedrich *et al*.^24^ reported that neutrophils accumulate in cerebral microvessels and infiltrate brain parenchyma within 10 min after induction of SAH in rats, while Yang *et al*.^25^ observed leukocyte adhesion to postcapillary venules within 10 min after SAH in an *in vivo* microscopy study of mice. These findings indicate that inflammatory processes induced by rapid neutrophil accumulation at the site of the subarachnoid haematoma may contribute to early cerebral hypoperfusion as well as delayed cerebral vasospasm.

The present study was designed to examine whether neutrophil-mediated inflammation contributes to early cerebral cortical hypoperfusion in the hours following SAH. To this end, we compared regional cerebral perfusion and oxidative stress at the haemorrhage site between model mice with a normal neutrophil count and those with neutropenia induced by anti-Ly6G antibody during the first 24 hours after SAH.

## Materials and Methods

### Ethics, animals, and housing conditions

The animal experiments were approved by the responsible animal care committee (Landesuntersuchungsamt Rheinland-Pfalz) and conducted in accordance with the German Animal Welfare Act (TierSchG). All applicable international, national, and institutional guidelines for the care and use of animals were followed.

Male C57BL/6N mice (Janvier, Saint-Berthevin Cedex, France; age, 11–12 weeks) were used for all experiments. The mice were housed under controlled environmental conditions (12-h light–dark cycle, 23 ± 1 °C, 55% ± 5% relative humidity) with free access to water and food (Altromin, Lage, Germany). As a general marker of well-being, body weight was determined daily during the experiments.

Quantitative data collection was conducted in a blinded fashion (animals and sample tubes were marked by numbers, without any identifiers of group allocation).

### Randomisation, and study design

Sixty-six animals were randomised to two groups: 30 animals were assigned for imaging of cerebral perfusion and 36 animals for biochemical analysis. Within each group, subsets were randomly assigned to receive experimental SAH or sham surgery and neutropenia induction or control treatment (described below). In animals assigned to the cerebral perfusion group, serial measurements of cerebral cortical perfusion were performed before the surgical procedure and 15 min, 3 h, and 24 h after induction of SAH or sham surgery together with determination of ICP. The mean arterial blood pressure was also determined daily in the cerebral perfusion group during the experiments using a non-invasive system (Coda mouse tail-cuff system; Kent-Scientific, Torrington, CT, USA). In animals assigned to the biochemistry group, cerebral perfusion and blood pressure were not analysed. Rather, transcardiac perfusion was performed 15 min, 3 h, or 24 h after induction of SAH to analyse the subarachnoid haematoma as described below.

In the cerebral perfusion group, 15 animals were randomised to receive neutropenia induction (described below) and 15 to the matched control subgroup. In both subgroups, six animals were randomised to sham surgery and nine to SAH induction (described below). Of the 36 animals assigned to the group for biochemical analysis, 18 were randomised to the control subgroup and 18 to neutropenia induction. In the biochemical analysis group, all 36 animals received SAH induction.

**Figure 1:**
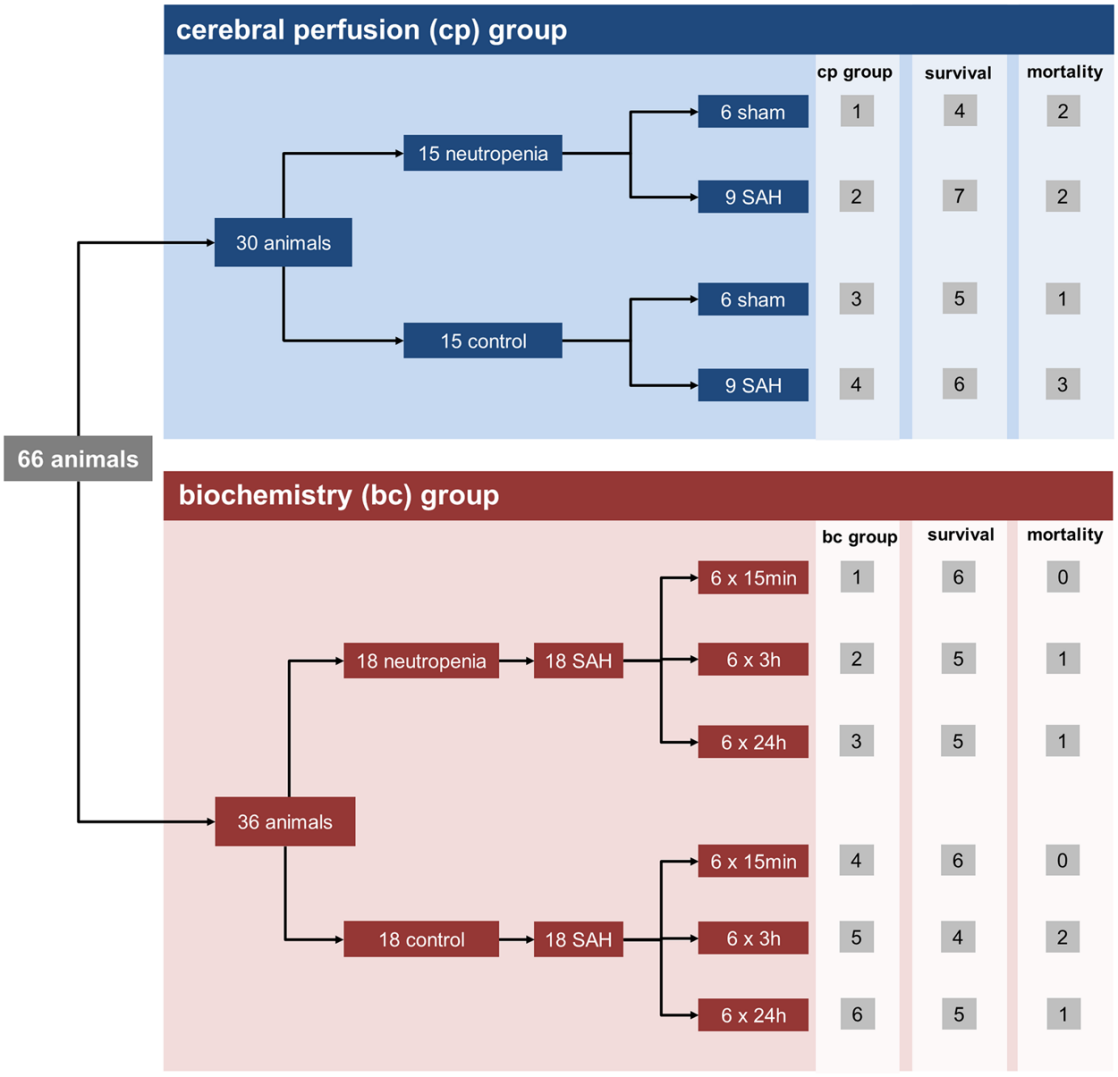
Overview of animal randomisation and mortality.

A detailed overview of animal randomisation is given in Fig. 1.

### Depletion of neutrophils, differential leukocyte count, and blood gas analysis

Intraperitoneal injection of an antibody directed against the Ly6G antigen, which is expressed by mature neutrophils and transiently by developing monocytes^26,27^, is an established method for the induction of neutropenia in mice^18,19^. In the neutropenia subgroups assigned for cerebral perfusion measurements or biochemical analysis, an anti-Ly6G antibody (InVivoPlus anti-mouse Ly6G, Clone 1A8; BioXCell, West Lebanon, NH, USA) was injected intraperitoneally at 40 µg/g body weight in 130 µl 12 h prior to SAH induction, while the corresponding control subgroups were injected with 130 µL of normal saline alone.

To analyse the efficacy of neutrophil depletion, blood samples were drawn from the left femoral artery before transcardiac perfusion for differential blood cell counting using an automated haematology analyser (ADVIA 2120; Siemens Healthineers, Erlangen, Germany). Counting and characterisation of murine leukocytes were performed using a veterinary software tool (VetMed version 6.3.2; Siemens Healthineers) that accounts for the haematological differences among murine species.

Blood gas analysis was performed on blood taken from the left femoral artery using a blood gas analyser (ABL 800 basic, Radiometer GmbH, Krefeld, Germany).

### Murine endovascular filament perforation model of SAH

Subarachnoid haemorrhage was induced by endovascular filament perforation under isoflurane anaesthesia (Abbvie, Wiesbaden, Germany) with continuous ICP monitoring as described previously^28–30^. Anaesthesia was induced with 4% isoflurane for 1 min and maintained with 2% isoflurane and 40% O_2_. For analgesia, Buprenorphin (Indivior, Slough, Berkshire, UK; 0.1 mg/kg body weight) was injected subcutaneously twice daily starting before the induction of SAH. Body temperature was kept constant at 36 °C using a heating pad (Physitemp Instruments LLC, Clifton, NJ, USA).

To induce SAH, anaesthetised mice were placed in the prone position and an ICP probe (Codman, Johnson & Johnson, Raynham, MA, USA) was placed through a burr hole in the right frontal region. After surgical preparation in the supine position, a filament (Prolene 5.0; Ethicon, Norderstedt, Germany) was inserted into the left external carotid artery and was advanced intracranially through the internal carotid artery^28–30^. A sharp rise in intracranial pressure was taken as an indicator of successful endovascular perforation. The ICP probe was removed at the end of the surgery. Wound closure was performed using Prolene 6.0 sutures (Ethicon). In sham animals, this surgical procedure was performed identically but without intracranially advancing the endovascular filament. After surgery, the animals were kept in an incubator heated to 36 °C (IC8000; Draeger, Luebeck, Germany) for 1 h to prevent hypothermia.

### Determination of cerebral cortical perfusion

Cerebral cortical perfusion was measured before SAH or sham surgery as well as 15 min, 3 h, and 24 h after surgery. All measurements were performed under isoflurane anaesthesia while keeping the body temperature at 36 °C using a heating pad (Physitemp Instruments LLC, Clifton, NJ, USA). Cerebral cortical perfusion was determined using a laser perfusion imager (MoorFLPI-2-blood flow imager; Cologne, Germany) and analysed with Moor review software (moorFLPI Full-Field Laser Perfusion Imager Review Version 4.0) as described^28^. In brief, the animal was mounted on a stereotaxic frame (Stoelting CO., Wood Dale, IL, USA). Following a midline incision to expose the calvaria, 60 transcalvarian perfusion images were recorded at 1 picture per second. The wound was then closed with Prolene 6.0 sutures and anaesthesia was terminated.

**Figure 2:**
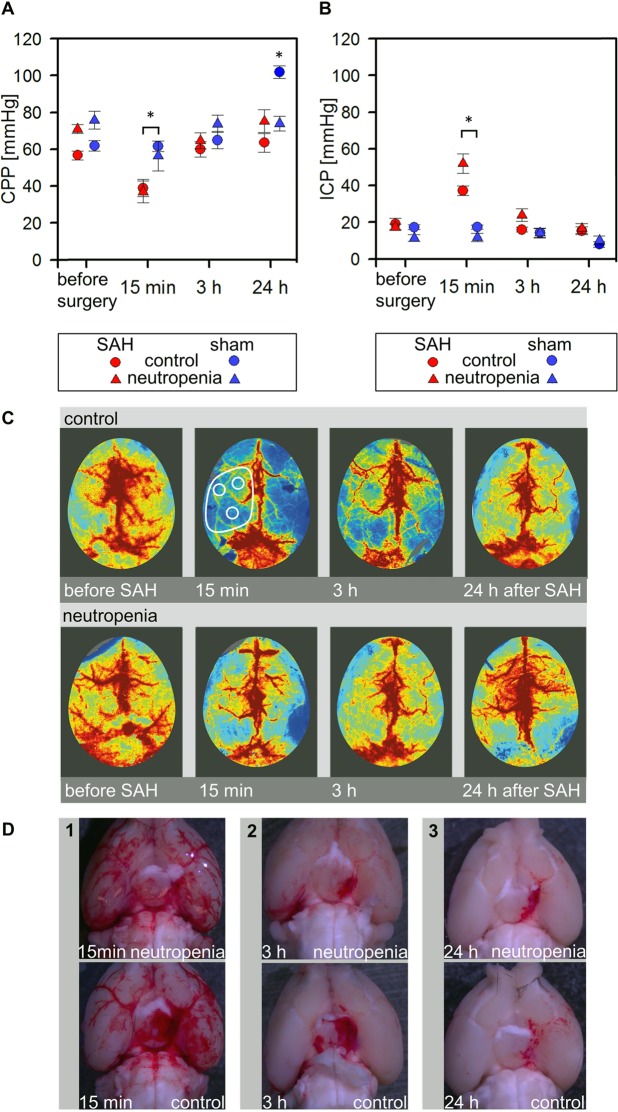
Murine model of subarachnoid haemorrhage. (**A**,**B**) Time courses of CPP and ICP changes after induction of SAH or sham surgery. Note that ICP increases minutes after SAH induction but returns to near baseline after 3 h, leading to a similar CPP as that before induction. (*p < 0.05; error bars indicate SEM; group sizes: SAH-neutropenia/cerebral perfusion group 2, n = 7; SAH-control/cerebral perfusion group 4, n = 6; sham-neutropenia/cerebral perfusion group 1, n = 4; sham-control/cerebral perfusion group 3, n = 5). (**C**) Representative images of cerebral cortical perfusion before and after induction of SAH in mice receiving vehicle or anti-Ly6G antibody to induce neutropenia. (**D**) Panels 1, 2, and 3 show representative images 15 min, 3 h, and 24 h after induction of SAH.

A mean image was calculated from the 60 perfusion images. The mean flux values were determined by evaluating three regions of interest (ROIs), each 2 mm in diameter, placed over the perfusion territory of the left middle cerebral artery as shown in Fig. 2. A mean flux value was calculated from the flux values determined over the three ROIs. Changes in perfusion are expressed relative to the preoperative flux value.

### Determination of 8-iso prostaglandin F2α content in the subarachnoid haematoma

Accumulation of 8-iso prostaglandin F2α (8-isoprostane), which is generated by peroxidation of arachidonic acid and arachidonic esters in phospholipids, is an established surrogate parameter for oxidative stress^31,32^. To analyse the effect of neutropenia on oxidative stress levels, 8-isoprostane levels were determined in basal subarachnoid haematomas isolated from mice assigned to the biochemical analysis group. Briefly, the mice were anaesthetised with isoflurane. After blood sampling for blood gas analysis and differential leukocyte count, transcardiac perfusion was performed with ice-cold Dulbecco’s Phosphate Buffered Saline containing MgCl_2_ and CaCl_2_, pH 7.4 (DPBS; Sigma-Aldrich, Hamburg, Germany) at 10 mL/min for 90 s. The brain was rapidly removed and immediately transferred to ice-cold DPBS. After photo documentation of the haematoma location (examples are shown in Fig. 2), the basal subarachnoid haematoma was isolated surgically, transferred to a safe-lock tube (Eppendorf, Wesseling-Berzdorf, Germany), and immediately shock-frozen in liquid nitrogen. The samples were stored at −80 °C until analysis.

The haematoma samples (approximately 1–2 mg wet weight) were homogenised in 50 μL antioxidant-supplemented lysis buffer (100 mM TRIS pH 7.4, 1 mM EDTA, 1 mM DTT, 10 μM phenothiazine) by sonication at low intensity for 3 × 5 s on ice^33^. The homogenates were then centrifuged at 8,000 × *g* for 10 min at 4 °C to pellet any particulate or fibrillar matter and the supernatants were transferred into clean tubes for protein and 8-isoprostane determination. The protein content was measured by BCA/copper reduction (Protein Assay Kit from Thermo Fisher Scientific, Rockford, IL, USA) and adjusted to 400 µg/mL with lysis buffer. The soluble 8-isoprostane concentration in the adjusted samples was then analysed by competitive enzyme immunoassay (8-isoprostane ELISA Kit; Cayman Chemical Company, Ann Arbor, MI, USA) following the manufacturer’s instructions.

### Gene expression analysis

RNA was isolated from the left hemispheres of brain samples using a Qiagen-RNeasy Plus Universal kit (Qiagen, Hilden, Germany) and reverse-transcribed into cDNA with QuantiTect Reverse Transcription Kit (Qiagen) according to the manufacturer’s instructions. The mRNA quantification by real-time quantitative polymerase chain reaction (qPCR) was performed as described^30,34–36^. Briefly, the cDNA of each sample was amplified using a real-time Lightcycler 480 PCR System (Roche). RT-PCR for the detection of cyclophilin A (PPIA), interleukin 1β (IL-1β), tumour necrosis factor α (TNFα), and inducible nitric oxide (NO) synthase (iNOS) was carried out using this system^30,34–36^. Absolute copy numbers of target gene mRNA expression were normalised by the absolute number of housekeeping gene copy numbers (PPIA) as described previously^30,34–36^. For normalisation, we used naive brain tissue of 5 C57bl6N mice not included in the cerebral perfusion or biochemistry study and which had not undergone any treatment.

### Statistics

Sigma Plot version 12.5 (Systat Software Inc., San Jose, CA, USA) was used for all statistical analysis. The data are presented as mean ± standard error of the mean (SEM). Group and subgroup means were compared by Mann–Whitney *U* test or with one-way ANOVA and HolmSidak multiple comparison testing as appropriate with p < 0.05 considered statistically significant. Group sizes were based on power analyses with Sigma Plot software set with an alpha of 0.05, power of 0.8, and expected group differences in perfusion values of 25% ± 13% between the groups based on pilot experiments. These calculations resulted in a group size of 6.

## Results

### Murine model of SAH

Endovascular filament perforation was followed by a prompt increase in ICP, indicating successful modeling of SAH. Moreover, a subarachnoid haematoma was present in all mice after endovascular filament perforation, whereas no subarachnoid haematomas were found in mice subjected to sham surgery. Details on the parameters collected during and after surgery are given in Figs 1 and 2 and in the supplementary information.

### Intraperitoneal injection of anti-Ly6G antibody induces neutropenia

Intraperitoneal injection of the anti-Ly6G antibody significantly reduced total leukocyte and neutrophil counts compared to vehicle injection. An evaluation of all 27 animals which received an anti-Ly6G injection to induce neutropenia, and all 26 animals which were injected with normal saline is shown in Table 1. Monocyte counts were also reduced by anti-Ly6G injection, in agreement with findings that Ly6G is a leukocyte lineage marker expressed during development of monocytes as well as by mature neutrophils^26,27^. In contrast, there were no significant differences in haemoglobin concentrations or lymphocyte, eosinophil, basophil, and platelet counts between anti-Ly6G-injected and vehicle-injected mice, confirming treatment specificity.

**Table 1:** Differential leukocyte counts between the control and neutropenia subgroups.

	neutropenia*	control*	p value
leukocytes/nL	2.74 ± 0.43	4.24 ± 0.47	p < 0.01
neutrophils/nL	0.60 ± 0.19	1.54 ± 0.19	p < 0.001
monocytes/nL	0.05 ± 0.01	0.16 ± 0.05	p < 0.001
lymphocytes/nL	1.73 ± 0.38	2.35 ± 0.44	n. s.
eosinophils/nL	0.09 ± 0.01	0.12 ± 0.02	n. s.
basophils/nL	0.01 ± 0.003	0.01 ± 0.001	n. s.
haemoglobin [g/dL]	14.4 ± 0.3	14.6 ± 0.3	n. s.
platelets/nL	1194 ± 44	1316 ± 63	n. s.

*The table summarises all blood samples from the cerebral perfusion and the biochemistry groups, i.e., blood samples of 27 animals with neutropenia and 26 animals without induction of neutropenia. Data are shown as mean ± SEM.

Cell counts were also evaluated separately for cerebral perfusion (11 animals with and 11 animals without neutropenia induction by anti-Ly6G injection) and the biochemistry groups (16 animals with and 15 animals without neutropenia induction by anti-Ly6G injection). There were also significant differences in cell counts within the cerebral perfusion group (*total leukocytes, anti-Ly6G:* 1.93 ± 0.53/nL *vs. vehicle:* 3.37 ± 0.51/nL, p < 0.05; *neutrophils:* 0.88 ± 0.46/nL *vs*. 1.86 ± 0.32/nL, p < 0.05; *monocytes:* 0.05 ± 0.01/nL *vs*. 0.21 ± 0.11, p < 0.05) and biochemistry group (*leukocytes, anti-Ly6G:* 3.27 ± 0.61/nL *vs. vehicle:* 4.91 ± 0.70/nL, n.s.; *neutrophils:* 0.41 ± 0.07/nL *vs*. 1.28 ± 0.20/nL, p < 0.001; *monocytes:* 0.05 ± 0.01/nL *vs*. 0.13 ± 0.02/nL, p < 0.001). Thus, the effect of anti-Ly6G antibody was reproducible and independent of the subsequent treatment.

### SAH enhances the expression of IL-1β, iNOS, and TNFα

To examine whether (i.) SAH induced expression of inflammation markers and (ii.) whether cerebral inflammation marker expression is susceptible to the induction of neutropenia, brain samples of the animals of the biochemistry group sacrificed after 24 h were taken to analyse markers of inflammation^30,34–36^. Compared with naive brain, SAH induction in the absence of neutropenia significantly enhanced the tissue expression of all three inflammation markers (IL-1β: 477 ± 99%; TNFα: 3978 ± 318%; iNOS: 150 ± 17%).

**Figure 3:**
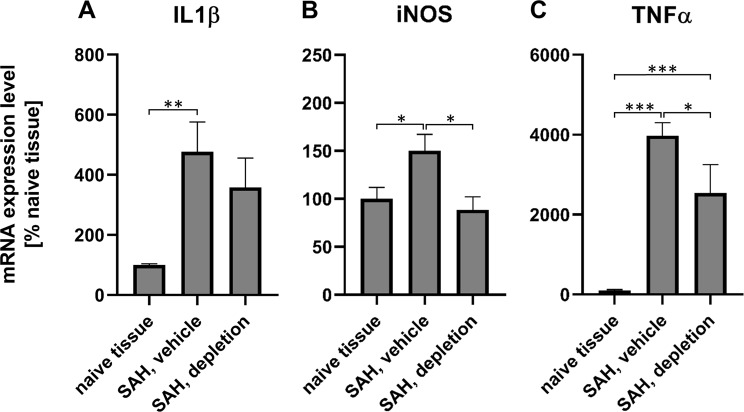
Gene expression of inflammation markers 24 h after SAH. Gene expression of IL-1β (**A**), iNOS (**B**), TNFα (**C**) 24 hours after induction of SAH (biochemistry groups 3 (n = 5) and 6 (n = 5), *p < 0.05; **p < 0.01; ***p < 0.001).

The induction of neutropenia attenuated the expression of all three inflammation markers, with a difference that reached statistical significance for iNOS and TNFα (SAH, neutropenia vs. normal neutrophil counts; iNOS: 89 ± 13% vs. 150 ± 17%, p < 0.05; IL-1β: 358 ± 98% vs. 477 ± 99%; TNFα: 2542 ± 711% vs. 3978 ± 318%, p < 0.05). These results indicate that the induction of neutropenia attenuated cerebral inflammation triggered by the induction of SAH (Fig. 3).

### Induction of neutropenia improves cerebral cortical hypoperfusion independent of CPP

#### Cerebral cortical perfusion

**Figure 4:**
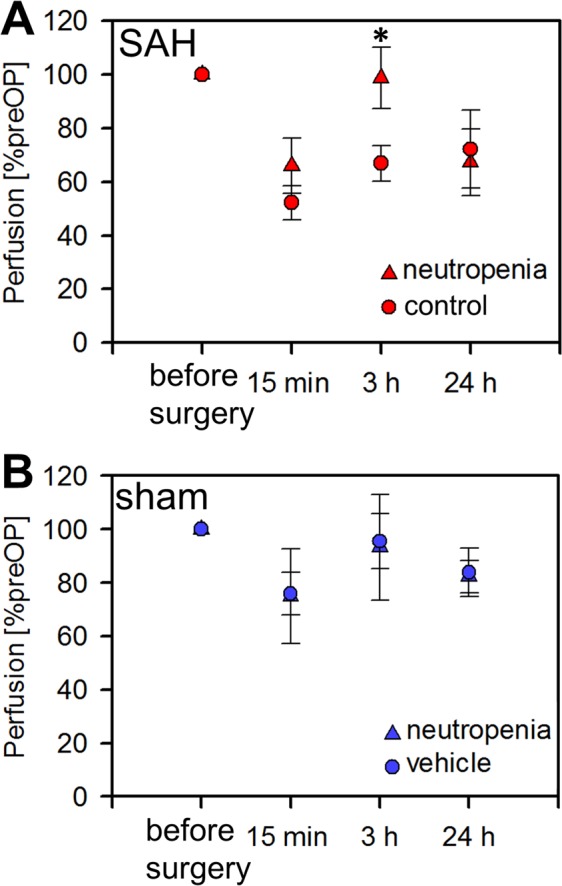
Enhanced cerebral cortical perfusion after SAH by induction of neutropenia. (**A**,**B**) Relative cerebral cortical perfusion after induction of SAH or sham surgery. (*p < 0.05; error bars indicate SEM; group sizes: SAH-neutropenia/cerebral perfusion group 2, n = 7; SAH-control/cerebral perfusion group 4, n = 6; sham-neutropenia/cerebral perfusion group 1, n = 4; sham-control/cerebral perfusion group 3, n = 5).

Cerebral cortical perfusion did not differ between the anti-Ly6G-treated (neutropenia) and vehicle-treated (control) subgroups either before or after sham surgery. Further, perfusion values did not differ between the vehicle-treated and anti-Ly6G-injected subgroups before SAH induction. In contrast, cerebral perfusion was markedly reduced after SAH surgery in the vehicle-injected subgroup compared to the vehicle-injected sham subgroup, while the flux values in the anti-Ly6G-injected (neutropenia) SAH subgroup were significantly higher than in the vehicle-injected SAH subgroup at 3 h post SAH (99% ± 11% *vs*. 65% ± 8%, p < 0.05). Thus, neutropenia appears to improve hypoperfusion following SAH. Figure 2 presents typical perfusion images in vehicle-treated (control) and anti-Ly6G-injected (neutropenia) subgroup mice. The mean results are shown in Fig. 4.

#### Cerebral perfusion pressure

CPP was calculated as the difference between the mean arterial pressure and the ICP determined before the measurements of cerebral perfusion. A significant difference in CPP between the SAH and sham mice was observed only at peak ICP immediately after endovascular filament perforation and 15 min after induction of SAH, but not before SAH induction or 3 h after. At 24 h after SAH, we observed a higher CPP in the sham/vehicle subgroup due to higher mean arterial pressure. The reason for this difference remains unclear. CPP did not differ among the other subgroups (Fig. 2**)**. Importantly, CPP did not differ 3 h after SAH between neutropenia and vehicle-injected control animals (*neutropenia:* 64.4 ± 4.8 mmHg *vs. control:* 60.0 ± 4.2 mmHg), so the significant improvement of cerebral perfusion at 3 h after SAH in neutropenic animals was not due to a difference in CPP.

#### Blood gases

Arterial blood samples were taken at the end of the experiments for evaluation of pO_2_, pCO_2_, and pH. There were no significant differences after induction of neutropenia or saline treatment (*pO*_2_*, neutropenia:* 144.7 ± 11.9 mmHg *vs. control:* 152.0 ± 10.7 mmHg; *pCO*_2_*, neutropenia:* 46.7 ± 1.5 mmHg *vs*. *control*: 44.4 ± 1.6 mmHg; *pH, neutropenia:* 7.3 ± 0.01 *vs. control:* 7.3 ± 0.01). Furthermore, blood gas values were similar between cerebral perfusion and biochemistry groups, and among the subgroups.

### Induction of neutropenia reduces oxidative stress in the subarachnoid haematoma

**Figure 5:**
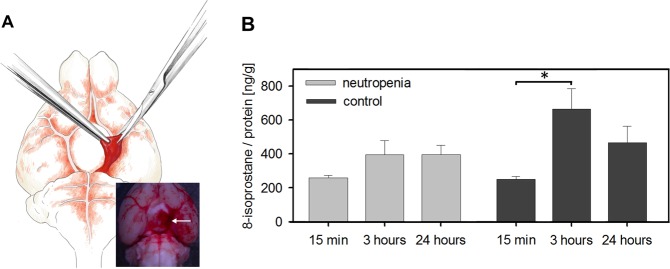
Reduced oxidative stress marker (8-isoprostane) accumulation in the subarachnoid haematoma of mice with induced neutropenia. (**A**) Isolation of the basal subarachnoid haematoma (drawing by Stefan Kindel, Mainz, Germany). (**B**) 8-isoprostane levels in the subarachnoid haematoma after induction of neutropenia or vehicle treatment. Note that the significant increase in the 8-isoprostane levels 3 h after SAH in the vehicle group was markedly attenuated in the neutropenia group. (*p < 0.05; error bars indicate SEM; group sizes: SAH-neutropenia (biochemistry groups 1–3): 15 min n = 6, 3 h n = 5, 24 h n = 5 ; SAH-control (biochemistry groups 4–6): 15 min n = 6; 3 h n = 4; 24 h n = 5).

Animals of the biochemistry group were sacrificed 15 min, 3 h, and 24 h after induction of SAH and the 8-isoprostane levels were measured in the basal subarachnoid haematoma as a surrogate parameter of oxidative stress (Fig. 5). In the vehicle-treated control subgroup, the 8-isoprostane levels per total protein rose significantly from 15 min to 3 hours after SAH and then decreased by 24 h. Induction of neutropenia markedly attenuated the increase in 8-isoprostane levels between 15 min and 3 h, suggesting that neutrophil accumulation in the haematoma contributes to oxidative stress.

## Discussion

To the best of our knowledge, this study is the first to investigate the impact of neutrophils on cortical perfusion in the initial phase after SAH. In animals subjected to experimental SAH by endovascular filament perforation, neutropenia was associated with significantly improved cerebral cortical perfusion at 3 h post-induction. In addition, neutropenia markedly reduced oxidative stress as measured by 8-isoprostane accumulation in the subarachnoid haematoma. Taken together, these findings indicate that neutrophils play a major role in cerebral hypoperfusion and oxidative stress in the early period after SAH, and strongly suggest that cerebral cortical hypoperfusion during the first hours after SAH is dependent on neutrophil-induced cerebral inflammation.

The pathophysiological processes leading to cerebral hypoperfusion in the early hours after SAH are not fully understood^4–6^. As demonstrated in the present and other experimental SAH studies^28,30,37–42^, subarachnoid bleeding elevates ICP, which in turn reduces CPP. ICP and CPP returned to near baseline levels within 3 hours after initial bleeding, in accordance with a report by Westermeier *et al*.^41^. However, despite sufficient CPP, cortical hypoperfusion persisted in SAH mice with normal neutrophil counts. In contrast, in SAH mice with neutropenia cortical perfusion was significantly higher compared to the SAH mice with normal neutrophil counts, indicating a role of neutrophils in mediating cerebral hypoperfusion in the hours after SAH.

A decrease in nitric oxide (NO) through NO scavenging appears to be a major mechanism leading to microvascular constriction and cerebral hypoperfusion in the early phase after SAH^38,39,43–45^. Reduced NO levels are believed to be a result of haemolysis and oxidative stress, leading to destructive redox reactions with NO. Neutrophil extravasation starts within minutes after SAH^24,25^. Upon activation, neutrophils release a cocktail of redox-active factors, such as myeloperoxidase. This enzyme acts as a potent oxidative stress inducer by generating hypochlorous acid^46^, which reacts with various biomolecules such as NO, leading to a reduction in local NO levels and impaired vasorelaxation. Isoprostanes occur after peroxidation of arachidonic acid and arachidonic esters in phospholipids under oxidative stress^31,32^. They were shown to regulate endothelial function and to induce vasoconstriction^47–49^. Here we directly demonstrated 8-isoprostane accumulation in the subarachnoid haematoma, which, besides the decrease in nitric oxide, may contribute to cortical hypoperfusion.

Cerebral hypoperfusion during the first hours after SAH also occurs in humans^8–14^. Moreover, these clinical studies showed that the extent of cerebral hypoperfusion early after SAH is strongly associated with DCI development and neurological outcome. Improving cerebral hypoperfusion early after SAH could therefore be a promising target for new therapeutic approaches. Current treatment strategies aim at optimising haemodynamic conditions and at controlling metabolic situation and ventilation^1–3^; however, a causal therapy directly modulating the pathomechanisms inducing cerebral hypoperfusion is not available. This study provides new pathophysiological data strongly suggesting that oxidative stress induced by neutrophils is a major trigger for vasoconstriction resulting in cortical cerebral hypoperfusion early after SAH. Modulation of neutrophil function or of neutrophil-induced oxidative stress could be a starting point for new therapies aiming at improving cerebral hypoperfusion early after SAH.

Finally, we want to address the limitations of this study. Clinical research data indicate that the degree of cerebral hypoperfusion during the first hours after SAH determines the severity of EBI, occurrence of DCI, and neurological outcome^4,8–14^. It would therefore be interesting to examine the effects of neutropenia on DCI and long-term neurological outcome. However, in our study, animals were already sacrificed after 15 min to 24 h. Therefore, the neutropenia effects on long-term neurological outcome and the incidence of DCI could not be analysed. It should be noted, however, that a beneficial long-term effect of neutropenia on vasospasm and neurological outcome has already been shown in a murine SAH model by Provencio *et al*.^18,19^. *Secondly*, neutrophils are an essential part of the innate immune system and neutropenia renders a patient susceptible to infections^21–23^. We therefore assume that therapies inducing neutropenia in SAH patients would induce severe complications, outweighing the beneficial effects of neutropenia on cerebral hypoperfusion. The approach chosen here to investigate the pathophysiological mechanisms underlying cerebral cortical hypoperfusion early after SAH is therefore not suitable for clinical applications. However, the novel pathophysiological insights on the importance of neutrophils may be used as a starting point to develop new therapeutic approaches. *Thirdly*, injection of the anti-Ly6G antibody induced not only neutropenia but also significantly reduced the number of monocytes. This fact is in agreement with findings that Ly6G is a leukocyte lineage marker expressed during the development of monocytes, as well as by mature neutrophils^26,27^. Monocytes have been reported to accumulate in the subarachnoid space two days after SAH^50^. Conversely, neutrophils have been reported to accumulate within minutes after SAH^21–23^. Although it therefore appears unlikely that monocytes play a major role in mediating cerebral cortical hypoperfusion early after SAH, we cannot exclude their contribution.

## Conclusion

Neutropenia significantly improved cortical cerebral hypoperfusion and reduced haematoma 8-isoprostane levels, a marker of oxidative stress, in the initial hours after SAH. The data therefore suggest neutrophils as an important mediator of cerebral hypoperfusion and oxidative stress in the early period after SAH. Although the experimental approach chosen here is not suitable for clinical applications, the findings of this study indicate that modulation of neutrophil function and neutrophil-induced oxidative stress could be a promising new approach to ameliorate cerebral hypoperfusion in the initial phase after SAH.

## Supplementary information

Supplementary Information

## Data Availability

The data that support the findings of this study are available from the corresponding authors upon reasonable request.
