# Supplementary material for: Neutrophils mediate early cerebral cortical hypoperfusion in a murine model of subarachnoid haemorrhage
Source: Sci Rep. 2019 Jun 11;9:8460. doi: 10.1038/s41598-019-44906-9 (PMC6560094; doi:10.1038/s41598-019-44906-9)
Supplement: Supplementary file 1 — Supplementary Information [file 41598_2019_44906_MOESM1_ESM.pdf]

Neutrophils mediate early cerebral cortical hypoperfusion in a murine model of subarachnoid haemorrhage

Axel Neulen*, Tobias Pantel, Michael Kosterhon, Andreas Kramer, Sascha Kunath, Maximilian Petermeyer, Bernd Moosmann, Johannes Lotz, Sven R. Kantelhardt, Florian Ringel, Serge C. Thal*

SUPPLEMENTARY INFORMATION

Results

Subarachnoid hematoma

All SAH animals, but none of the sham mice, developed subarachnoid hematoma. In all brain samples collected at 15 min after SAH, we observed a pronounced basal subarachnoid hematoma as well as a perivascular subarachnoid hematoma surrounding the ipsilateral and contralateral cerebral arteries and their cortical branches. In brain samples taken 3 and 24 h after induction of SAH, the basal subarachnoid hematoma was present in all samples, but was smaller compared to brain samples removed 15 min after SAH. Typical brain samples are shown in **Figure 2** of the main manuscript.

Mortality

Altogether, 13 mice died, 10 SAH mice (seven after induction of neutropenia and three after vehicle treatment) and three sham mice (two after induction of neutropenia and one after vehicle treatment). These animals were omitted from further analysis. Mortality in SAH mice was higher compared to that in sham animals, and among the SAH mice, mortality was higher in the cerebral perfusion group compared to the biochemistry group. In the cerebral perfusion group, five SAH mice died (three after induction of neutropenia and two after vehicle treatment) and three sham mice died (two after induction of neutropenia and one after vehicle treatment). In the biochemistry group, five SAH mice died, two after induction of neutropenia (one assigned to transcardiac perfusion after 3 h and one assigned to transcardiac perfusion after 24 h) and three after vehicle treatment (two assigned to transcardiac perfusion after 3 h, and one assigned to transcardiac perfusion after 24 h). The higher mortality in the cerebral perfusion group is explained by the fact that the majority of the animals in the

biochemistry group were sacrificed 15 min and 3 h after surgery, while all animals in the cerebral perfusion group were observed over 24 h. **Figure 1** of the main manuscript gives an overview of the different groups and mortality.

Parameters collected during surgery

In the cerebral perfusion group, the mean duration of the surgery was similar between the SAH (47 ± 2 min) and the sham (42 ± 3 min) subgroups. In the biochemistry group, the duration of SAH surgery was shorter (30 ± 1 min) because these animals did not undergo the imaging procedure for cerebral perfusion. Within both the cerebral perfusion and biochemistry groups, the duration of surgery was similar between animals subjected to neutropenia induction or vehicle treatment.

Body weight

The loss of body weight was similar between the SAH and sham animals in the cerebral perfusion group (before surgery: SAH 25.8 ± 0.4 g, sham 25.2 ± 0.5 g; postop day 1: SAH 23.5 ± 0.3 g, sham 22.7 ± 0.5 g) and between animals subjected to neutropenia or vehicle injection. There were also no significant differences between SAH animals in the cerebral perfusion group and those in the biochemistry group that survived 24 hours after SAH induction (before surgery: SAH 25.6 ± 0.5 g; postop day 1: SAH 22.3 ± 0.5 g), or between animals subjected to neutropenia induction or vehicle injection.
